# Complete genome sequence of community-associated methicillin-resistant *Staphylococcus aureus* (strain USA400-0051), a prototype of the USA400 clone

**DOI:** 10.1590/0074-02760170128

**Published:** 2017-11

**Authors:** Marina Farrel Côrtes, Maiana OC Costa, Nicholas CB Lima, Rangel C Souza, Luiz GP Almeida, Luciane Prioli Ciapina Guedes, Ana TR Vasconcelos, Marisa F Nicolás, Agnes MS Figueiredo

**Affiliations:** 1 Universidade Federal do Rio de Janeiro, Instituto de Microbiologia Paulo de Góes, Laboratório de Biologia Molecular de Bactérias, Rio de Janeiro, RJ, Brasil; 2 Laboratório Nacional de Computação Científica, Petrópolis, RJ, Brasil

**Keywords:** methicillin-resistant *Staphylococcus aureus*, whole genome, USA400, ST1-SCC*m*ecIV

## Abstract

*Staphylococcus aureus subsp. aureus*, commonly referred as *S. aureus*, is an important bacterial pathogen frequently involved in hospital- and community-acquired infections in humans, ranging from skin infections to more severe diseases such as pneumonia, bacteraemia, endocarditis, osteomyelitis, and disseminated infections. Here, we report the complete closed genome sequence of a community-acquired methicillin-resistant *S. aureus* strain, USA400-0051, which is a prototype of the USA400 clone.

Methicillin-resistant *Staphylococcus aureus* (MRSA) isolates from the ST1-SCCmecIV lineage are regularly associated in healthy community-dwelling individuals. These isolates were first reported in Australia and named as Western Australia-1 clone (Udo et al. 1993). Later, ST1-SCC*mec*IV MRSA carrying *lukSF-PV* genes encoding the Panton-Valentine leucocidin (PVL) were detected in the USA and caused severe infections among American Indian children. The complete closed genome sequence of a representative of this MRSA, MW2, is deposited in GenBank (Baba et al. 2002). Years later, ST1-SCC*mec*IV MRSA (PVL^+^) re-emerged in the USA and Canada as an important cause of skin-soft tissues infections and was renamed as USA400 (McDougal et al. 2003, Golding et al. 2011). Here, we report the complete closed genome sequence of strain USA400-0051, a prototype of the USA400 clone, isolated during CA-MRSA outbreaks (2003-2006) of skin and soft tissue infections in the USA (Tenover et al. 2006).

Genomic DNA was obtained (Sambrook et al. 1989), and its concentration and purity were assessed using a Qubit^®^ 2.0 fluorometer (Invitrogen, Carlsbad, CA, USA). The library was prepared with 100 ng DNA. Adapter ligation, size selection, nick repair, and amplification were performed using the Ion Xpress Fragment Library Kit (Ion Torrent; Thermo Fisher Scientific, Waltham, MA, USA). The Ion Sequencing Kit v2.0 was used for all sequencing reactions following the manufacturer's recommendations. Torrent Suite 1.5 was used for the analysis, and sequencing was performed using 316 chips. The assembly, based on 741,383 reads, was carried out using Newbler v 2.6 (Roche Diagnostics, Basel, Switzerland) and Celera genome assembly v 6.1 (JCV Institute; Myers et al. 2000). Gaps within the scaffolds were resolved using genome sequencing performed using the 454 GS FLX titanium (3-kb paired-end library) approach (Roche). The genome was annotated using Sabia (Available from: www.sabia.lncc.br/; last access in August 2016) pipeline.

The genome of USA400-0051 consists of one circular chromosome with 2,832,530 base pairs (bp) and a GC content of 30.58%. A total of 2773 protein-coding sequences were annotated (2299: known functions, 392: unknown categories, and 81: pseudogenes). The genome harbours 16 rRNA genes (five copies of 16S rRNA, five of 23S rRNA, and six of 5S rRNA) and 40 tRNA genes, identified using RNAmmer and tRNAscan-SE (Schattner et al. 2005), respectively. The genome also contains a 2064-bp plasmid with a GC content of 28.75%.

The USA400-0051 strain was typed as *spa* t128 and SCC*mec*IVa (*ccrA2* and *ccrB2*). This strain harbours most of the *S. aureus* adhesion- and biofilm-associated genes: *ebh, clfA, clfB, cna, ebp, map, efb, fnbAB, icaABCDR, sdrCDE*, and *spa*, in addition to *atl*. The genome of USA400-0051 also contains an arsenal of enterotoxin and enterotoxin-like genes such as *sea, sec, seh*, and *sek* and *selq selk*, and *sell*, respectively, in addition to other toxin-associated genes including *sak, hla, hlb, hld, hlgA, hlgB, hlgC, snc, eta, lukSF*-PV, *lukDE,* and *lukXY* (encoding staphylokinase, α-, β- and δ-hemolysins, staphylococcal bi-component γ-hemolysin A, B, and C, staphylococcal complement inhibitory precursor, exfoliative toxin A, and the leucocidins Panton-Valentine-PVL, LukDE and LukXY; respectively). These virulence genes are mostly present in mobile genetic elements such as bacteriophages [(i) PhiSa2mw carrying the PVL genes (*lukSF*) and (ii) PhiSa3mw containing *sea, selk*, and *selq*] and genomic islands [(GI) (i) SAPImw2 carrying *ear, sec,* and *sell;* (ii) a type II vSAα carrying the gene clusters encoding staphylococcal superantigens *set16, set17, set18, set19, set20, set21, set22, set23, set24, set25,* and *set26* and lipoproteins *lpl10, lpl11, lpl12, lpl13,* and *lpl14*; and (iii) a type II vSAβ with an incomplete operon of the serine proteases *splABCF* and the lantibiotics epidermin locus *bsaA1, bsaA2, bsaB bsaD, bsaP, bsaF, bsaE,* and *bsaG*] (Fig. 1). Analyses using ResFinder 2.1 (www.cge.cbs.dtu.dk/services/ResFinder; last access in January 2016) showed that USA400-0051 is only resistant to β-lactam drugs and contains the genes *blaZ* and *mecA;* this was confirmed by an antimicrobial disc susceptibility test. A tree was built based on the 16S rRNA genes with RDP Tree Builder (www.rdp.cme.msu.edu/treebuilder/treeing.spr; last access in June 2016), using Weighbor with an alphabet size of four and length of 1000. The bootstrapping process was repeated 100 times to generate a strict consensus tree (Cole et al. 2007). Fig. 2 shows the phylogenetic position of USA400-0051 in relation to other *Staphylococcus* sp. genomes. The phylogenetic tree showed that USA400-0051 clustered with MW2 (the reference strain). The MW2 and USA400-0051 genomes showed few differences. Partial *sasG* and *cna* genes were present in the USA400-0051 genome, which lacked a 560-bp fragment for *cna* and a 1922-bp fragment for *sasG* (Fig. 3A-B). In addition, MW2 harbours one additional copy of 16S and 23S rRNAs compared to USA400-0051.

**Fig. 1:**
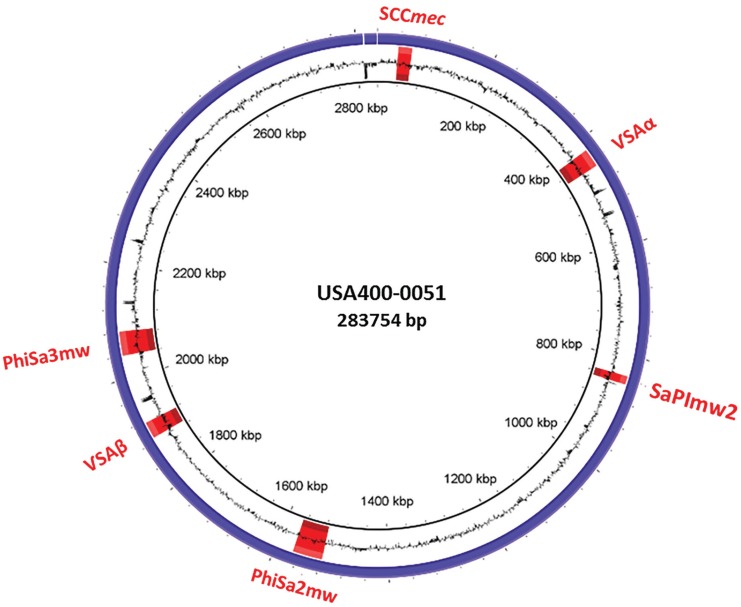
genomic atlas of strain USA400-0051 created using BRIG. The blue circle represents the genomic sequence and the black circle indicates the GC content. In red are the positions of the genomic island SCC*mec,* vSAα, SaPImw2, and νSAβ and bacteriophages PhiSa2mw and PhiSa3mw.

**Fig. 2:**
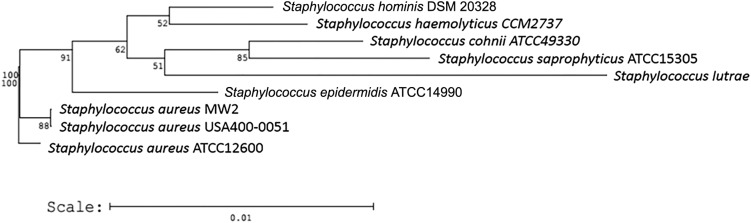
phylogenetic tree. ATCC 12600 (NCBI access: L36472), ATCC 15305 (NCBI access: AP008934), ATCC 14990 (NCBI access: D83363), DSM 20328 (NCBI access: X66101), CCM2737 (NCBI access: X66100), ATCC 49330 (NCBI access: AB009936). *Staphylococcus lutrae* sequence (NCBI access: X84731) was used as the outgroup.

**Fig. 3:**
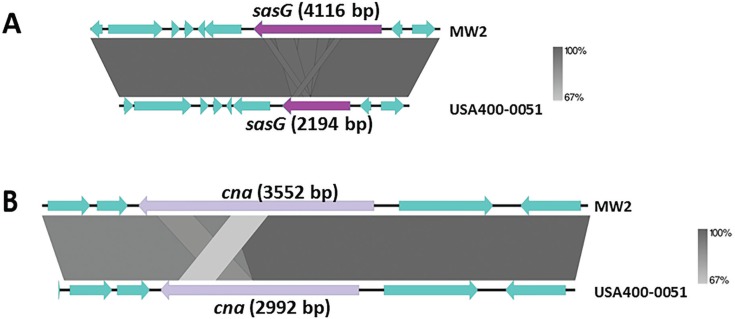
alignment of the context region comprising the genes sasG (A) and csn (B) in strain USA400-0051 and closely related MW2 strain.

The complete genome sequence of the USA400-0051 strain was deposited in the GenBank (WGS database) under NCBI accession number (chr: CP019574; plm: CP019575).
